# Enhanced inter-subject brain computer interface with associative sensorimotor oscillations

**DOI:** 10.1049/htl.2016.0073

**Published:** 2017-02-20

**Authors:** Simanto Saha, Khawza I. Ahmed, Raqibul Mostafa, Ahsan H. Khandoker, Leontios Hadjileontiadis

**Affiliations:** 1 Department of Electrical and Electronic Engineering, United International University, Dhaka, Bangladesh; 2 Electrical and Electronic Engineering Department, The University of Melbourne, Parkville, VIC, Australia; 3 Biomedical Engineering Department, Khalifa University of Science, Technology and Research, Abu Dhabi, UAE; 4 Department of Electrical and Computer Engineering, Aristotle University of Thessaloniki, Thessaloniki, Greece; 5 Department of Electrical and Computer Engineering, Khalifa University of Science, Technology and Research, Abu Dhabi, UAE

**Keywords:** brain-computer interfaces, handicapped aids, electroencephalography, biomedical electrodes, medical signal processing, signal classification, enhanced intersubject brain computer interface, associative sensorimotor oscillations, electroencephalography, EEG, electrophysiological signatures, cortical events, scalp, high-dimensional electrode montages, actual event related sources, intersubject variability, brain dynamics, psychophysiological states, wavelet coherence analysis, motor imagery, BCI, classification accuracy, brain sensorimotor regions, sensorimotor oscillations

## Abstract

Electroencephalography (EEG) captures electrophysiological signatures of cortical events from the scalp with high-dimensional electrode montages. Usually, excessive sources produce outliers and potentially affect the actual event related sources. Besides, EEG manifests inherent inter-subject variability of the brain dynamics, at the resting state and/or under the performance of task(s), caused probably due to the instantaneous fluctuation of psychophysiological states. A wavelet coherence (WC) analysis for optimally selecting associative inter-subject channels is proposed here and is being used to boost performances of motor imagery (MI)-based inter-subject brain computer interface (BCI). The underlying hypothesis is that optimally associative inter-subject channels can reduce the effects of outliers and, thus, eliminate dissimilar cortical patterns. The proposed approach has been tested on the dataset IVa from BCI competition III, including EEG data acquired from five healthy subjects who were given visual cues to perform 280 trials of MI for the right hand and right foot. Experimental results have shown increased classification accuracy (81.79%) using the WC-based selected 16 channels compared to the one (56.79%) achieved using all the available 118 channels. The associative channels lie mostly around the sensorimotor regions of the brain, reinforced by the previous literature, describing spatial brain dynamics during sensorimotor oscillations. Apparently, the proposed approach paves the way for optimised EEG channel selection that could boost further the efficiency and real-time performance of BCI systems.

## 1 Introduction

As an unconventional communication pathway, brain computer interface (BCI) enables us to communicate with a computer or with other external devices without any muscular stimulation. Although the primitive goal of developing BCI was to assist physically disabled people experiencing motor function abnormalities, recent technological advancements augment BCI in many other applications, including lie detection [1], brain fingerprinting [2], mood assessment [3] and gaming [4]. Most of the proposed BCIs are subject-specific and require time-consuming, sometimes frustrating calibration sessions. Thus, inter-subject BCIs are desired; yet development of such BCIs come across challenges including the inherent variabilities in brain dynamics across subjects due to the diversity in individual brain growth [5]. Moreover, multichannel electroencephalogram (EEG) that captures the electrical activity of the brain, suffers from the effect of outliers due to excessive channels, causing, at the same time, high computational burden to the BCI system. However, developing an inter-subject BCI with subjects who share associative neural oscillations for particular cognitive task, seems more feasible. Previous studies addressed inter-subject association of neural dynamics during natural vision [6] and natural music listening [7]. During motor imagery (MI)-based inter-subject BCI, it is important to measure sensorimotor synchronisation across subjects. MI is the kinesthetic imagination of a motor task, which shares equivalent sensorimotor oscillations corresponding to actual motor execution [8]. Movement-related cortical potential-based BCI without subject-specific training has been proposed in [9]. In [10], an inter-subject BCI has been developed for modifying mental states that can be used for treating major depressive disorder. Rana *et al.* [11] have developed a real-time toolbox for implementing inter- and intra-subject BCI using functional magnetic resonance imaging. An online inter-subject BCI with P300 speller paradigm has shown the deviation of inter-subject evoked potentials [12]. Learning from subspaces that have been estimated via common spatial pattern (CSP) applied on inter-subject/session data of similar characteristics, i.e. non-stationarities, can enhance the performance of BCI [13, 14]. In these experiments, selecting suitable subjects is critical due to the fact that the brain dynamics significantly vary across subjects. However, these methods perform well, specifically in the context of small training trials available from the target subject [15, 16]. Another study has proposed ensemble of classifiers, which can be used for single-trial classification without explicitly being trained [17]. In [18], sparse common spatial pattern is proposed as a novel method for optimal channel selection technique within a constraint of optimal classification accuracy. In this Letter, a coherence analysis in time–frequency (T–F) space is proposed to select the set of EEG channels, who have relatively high normalised coherence power. The underlying hypothesis is that highly coherent and common inter-subject EEG channels can improve BCI performances, by reducing the effect of outliers from undesired channels. To achieve this, the wavelet coherence (WC) has been adopted as a means to measure coherence between two time series in T–F domain, since it has successfully being used as a tool of measuring couplings between brain regions using sensory-evoked potentials [19] and to the associativity assessment of inter-personal brain activity [20].

## 2 Methods

At first, the available multichannel EEG data (Section 2.1) were preprocessed using CSP [21]. Then, wavelet decomposition (up to the third level) was applied, using the Daubechies three-sample filter (db3). At each decomposition level, subband energy and subband entropy were used as features for the classification of the MI types, which was realised via a two-layer feed-forward neural network [22] and used to classify MIs.

WC was estimated to discern if the coherent power reveals common inter-subject regions, where the sensorimotor oscillations from two subjects co-vary. Based on the WC power (WCP), some channels were selected assuming that those channels will enhance the T–F synchronisation of two subjects. Then, a novel inter-subject BCI framework was proposed with a view to validate whether selected channels can improve the classification performances. The motivation behind was to eliminate undesired channels which introduce outliers. On the contrary, selected channels enhance sensorimotor coherence; thus, the pairwise inter-subject BCI performances.

### 2.1 Dataset and experimental settings

**Fig. 1:**
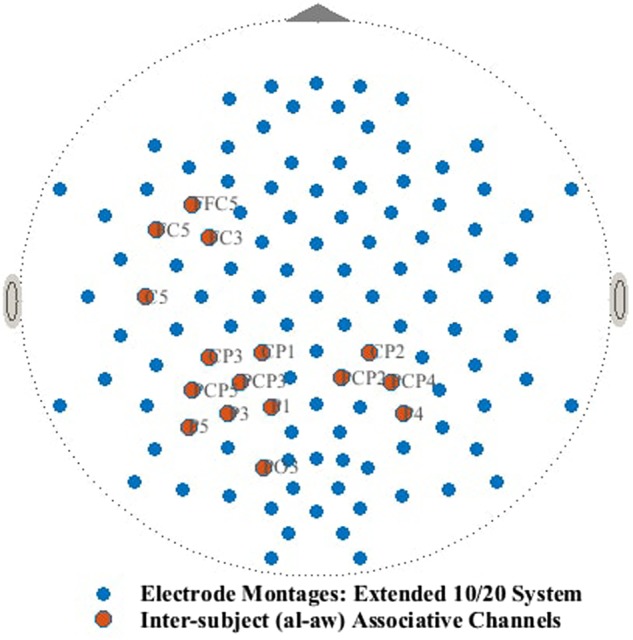
Electrode montages (extended 10/20 system) and selected inter-subject associative channels for subject air al-aw

The multichannel EEG raw signals used for the validation of the proposed hypothesis are those included in the dataset IVa from BCI Competition III (http://www.bbci.de/competition/iii/desc_IVa.html). One hundred and eighteen channels in total (extended 10/20 system) were employed to capture the multichannel EEG signals (Fig. 1). During the recording session, the sampling rate was set at 1kHz, which was later downsampled to 100 Hz. While sitting on a comfortable chair with arms resting on armrests, subjects were asked to perform each one of the three MIs, i.e. left hand, right hand (RH) and right foot (RF); the publicly provided data, however, refer to two MI classes only, i.e. RH and RF. Single-trial EEG data were captured from five healthy individuals, by providing them class-specific visual cues before the kinesthetic imagination of the motor tasks. The data consist of 280 trials (140 trials for each MI class) for each subject and each trial has 3.5 s of EEG recordings for any of the two MIs. It should be noted that 2.5 s of data for each trial after 1 s of the corresponding visual cues were chosen for analysis.

The proposed BCI framework is studied in pairwise manner, i.e. signals from one subject are used to train and validate the algorithm whereas signals from another subject are used to test the algorithm. Thus, there are total of 560 trials for experimenting inter-subject BCI. The first 180 trials are used to train the classifier, whereas the consecutive 100 trials are used to validate it, so to avoid over-fitting. Finally, the rest 280 trials from another subject are used to test the classifier.

### 2.2 Common spatial pattern

In 2000, Ramoser *et al.* proposed CSP, as a spatial filtering method, for classifying two classes of single-trial MIs for the first instance [21]. Usually, CSP estimates projection of multichannel EEG signal, so that the difference between two classes is maximised. Suppose $C_{\rm H}$ and $C_{\rm F}$ represent the two MIs, i.e. RH and RF, respectively, which have been considered in this experiment. Then, CSP approximates a weight matrix ***W*** that can be used to filter simultaneously recorded multichannel data. Estimating ***W*** requires simultaneous diagonalisation of two covariance matrices for two classes as follows

(1) $$\left\{{\matrix{ {W^{\rm T}\sum\limits_{C_{\rm H}} {{\bi W} = {\bf \Lambda }_{C_{\rm H}}} } \cr {W^{\rm T}\sum\limits_{C_{\rm F}} {{\bi W} = {\bf \Lambda }_{C_{\rm F}}} } \cr } } \right.\eqno\lpar 1\rpar $$

where $\sum\nolimits_{C_{\rm H}} $ and $\sum\nolimits_{C_{\rm F}} $ represent average covariance matrices over the training trials, whereas ${\bf \Lambda }_{C_{\rm H}}$ and ${\bf \Lambda }_{C_{\rm F}}$ are the diagonal matrices that must satisfy ${\bf \Lambda }_{C_{\rm H}} + {\bf \Lambda }_{C_{\rm F}} = {\bi I}$, where ***I*** is the identity matrix.

Finally, the original EEG signals can be projected as

(2) $$E_{{\rm csp}} = {\bi W}E\eqno\lpar 2\rpar $$

where *E* and $E_{{\rm csp}}$ represent the EEG signal and spatially filtered EEG signal, respectively, both having *N* number of channel components and each component constitutes of *P* number of samples per trial. While the first components of $E_{{\rm csp}}$ attribute to maximal discriminative features for $C_{\rm H}$ and minimal for $C_{\rm F}$, the last components of $E_{{\rm csp}}$ attribute to minimal discriminative features for $C_{\rm H}$ and maximal for $C_{\rm F}$. More details on CSP can be found in [21].

### 2.3 Wavelet coherence and channel selection

Suppose *x*(*t*) represents a time-domain signal, where $\lpar x\lpar t\rpar \in L^2\lpar {\opf R}\rpar \rpar $. Then, the continuous wavelet transform is defined as [23]

(3) $$W^x\lpar a\comma \; b\rpar = \displaystyle{1 \over {\sqrt a }}\int_{ - \infty }^\infty x\lpar t\rpar \psi ^\ast \left({\displaystyle{{t - b} \over a}} \right)\, {\rm d}t\eqno\lpar 3\rpar $$

where * represents the complex conjugate and $\psi \lpar t\rpar $ is the wavelet basis function (mother wavelet). Usually, $\psi \lpar t\rpar $ is dilated by a factor *a*, *a* > 0, whereas translated by a factor *b*, and these time and scale parameters (*a*, *b*) are continuous. In this study, the complex Morlet wavelet was adopted as the mother wavelet given by [23]

(4) $$\psi \lpar t\rpar = \displaystyle{1 \over {\sqrt {\pi f_{\rm b}} }}{\rm e}^{ - t^2/f_{\rm b}}\, {\rm e}^{{\rm j}2\pi f_{\rm c}t}\comma \; \eqno\lpar 4\rpar $$

where $f_{\rm b}$ is a bandwidth parameter and $f_{\rm c}$ is the wavelet centre frequency. As a Gaussian-windowed complex sinusoid, the adopted complex Morlet wavelet with its second-order exponential decay results in very good time localisation during the wavelet transform [23]. Additionally, this wavelet basis function gives information about both amplitude and phase, thus it is better used for capturing coherence between oscillatory harmonics in T–F space [23]. The values of $f_{\rm b}$ and $f_{\rm c}$ were set to 1 and 0.9549 Hz, respectively.

$W_n^{xy} $ is the cross wavelet transform (XWT) which finds the T–F regions where two signals co-vary, but have high power in contrast to WC. Suppose $x_n$ and $y_n$ are two time signals (where *n* = 1, 2, …, *N*), then the XWT is defined as

(5) $$W_n^{xy} \lpar s\rpar = W_n^x \lpar s\rpar \cdot W_n^{y^\ast } \lpar s\rpar \eqno\lpar 5\rpar $$

where *s* represents scale that is used to stretch the mother wavelet along time.

WC reveals associativity of two time-domain signals in T–F spaces. This similarity measure finds the T–F locations where the signals significantly co-vary, but does not necessarily have high power. The WC is defined as follows [24]

(6) $$R_n^2 \lpar s\rpar = \displaystyle{{\vert S\lpar s^{ - 1}W_n^{xy} \lpar s{\rpar \rpar \vert }^2} \over {S\lpar s^{ - 1}\vert W_n^x \vert ^2\rpar S\lpar s^{ - 1}\vert W_n^y \vert ^2\rpar }}\eqno\lpar 6\rpar $$

where *S* is a smoothing operator and defined as

(7) $$S\lpar W\rpar = S_{{\rm scale}}\lpar S_{{\rm time}}\lpar W_n\lpar s\rpar \rpar \rpar \eqno\lpar 7\rpar $$

**Fig. 2:**
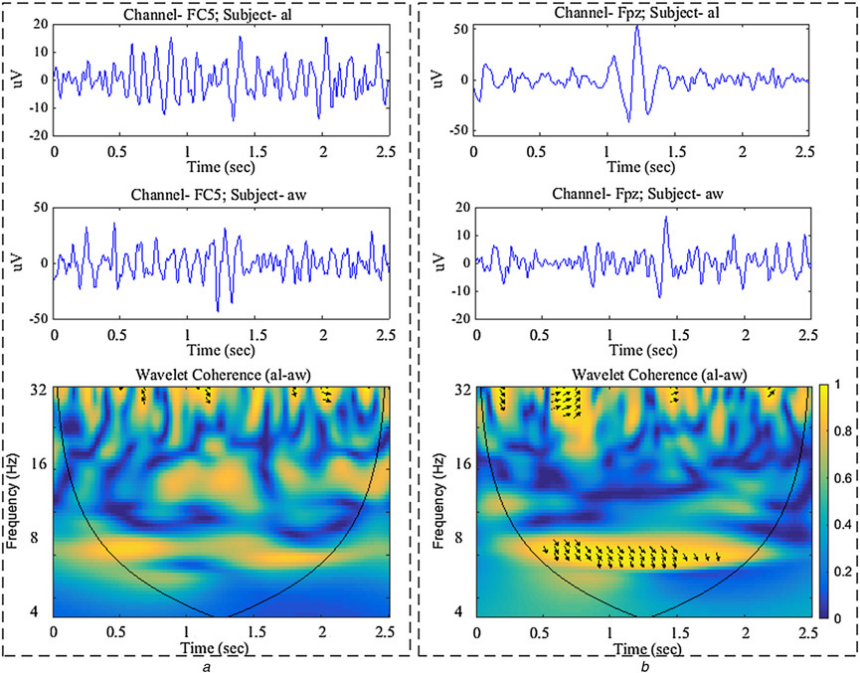
Calculation of WC between inter-subject sensorimotor oscillations (i.e. regarding RH MI task) for two different channels corresponding to subject pair al-aw *a* The channel FC5 has the highest $\hbox{WC}\hbox{P}_N$ value *b* The channel Fpz has the lowest $\hbox{WC}\hbox{P}_N$ value. The arrows in T–F WC plot indicate the phase relationship of two time series having WCP > 0.9. The range of $0.6 \le {\rm WCP} \le 0.9$ has only been considered significant while measuring inter-subject sensorimotor coherence

$S_{{\rm scale}}$ and $S_{{\rm time}}$ represent smoothing along scale/frequency and time axes, respectively. Fig. 2 shows the calculation of WC between two electrophysiological time series.

The single-trial EEG signals from two subjects have been considered as two sets. From each set, the trials are further categorised according to classes, i.e. RH or RF. Consider *S*1 and *S*2 as two individuals, each having total *M* trials and *M*/2 trials for each class. The WC has been applied to measure similarities of *M*/2 trials from *S*1 with *M*/2 trials from *S*2 one by one. The WCP is normalised to 1 and thus the average WCP ranges from 0 to 1. The accumulated WCP over trials are averaged and denoted as $\hbox{WC}\hbox{P}_N$, given by

(8) $$\hbox{WC}\hbox{P}_{Nc} = \displaystyle{2 \over M}\sum\limits_{i = 1}^{M/2} \lpar \lpar R_n^2 \lpar s\rpar \rpar _c\rpar _i\eqno\lpar 8\rpar $$

where $c \in \lpar {\rm RH}\comma \; {\rm RF}\rpar $. The $R_n^2 \lpar s\rpar $ in the range from 0.6 to 0.9 has been considered significant and used to measure associativity of inter-subject channels, i.e. $R_n^2 \lpar s\rpar \to \lsqb 0.6\comma \; 0.9\rsqb $. The implicit assumption is that very high coherence power (>0.9) might represent resting EEG dynamics, irrespective of any information regarding cortical events.

Finally, the estimated $\hbox{WC}\hbox{P}_N$ is used to rank the channels in descending order. Eight channels were selected for each class, accumulating to 16 channels for two classes. If any channel falls within the selection criteria for both classes, we have selected another channel with higher $\hbox{WC}\hbox{P}_N$ either for RH or RF, thus limiting the number of selected channels to 16. The selected channels are specified in Table 1.

**Table 1:** Selected associative inter-subject channels for different subject pairs along with corresponding *WC*P_N_

S1–S2	Selected channels
aa-al	RH$ \to $**C1**(0.207) CCP3(0.203) P1(0.201) FFC8(0.201) FFC7(0.201) F7(0.200) **FT10**(0.200) Pz(0.200) PCP1(0.199) RF$ \to $CCP8(0.210) C6(0.207) O1(0.204) T8(0.201) Oz(0.199) C1(0.199) FT10(0.199) PCP8(0.199) PO4(0.199)
aa-av	RH$ \to $P7(0.203) PPO7(0.202) P6(0.201) CP6(0.201) F6(0.201) Fz(0.201) PCP7(0.200) P9(0.200) RF$ \to $CP3(0.203) FT8(0.201) FC2(0.201) CFC1(0.200) C6(0.200) OI2(0.200) FC4(0.200) CCP6(0.199)
aa-aw	RH$ \to $CFC3(0.195) FT10(0.194) CFC6(0.194) CCP5(0.193) CCP8(0.193) CCP1(0.192) C6(0.192) **PCP1**(0.191) RF$ \to $CP4(0.210) C1(0.207) **PCP1**(0.207) PCP2(0.207) PCP4(0.207) CP6(0.207) FFC2(0.207) Fz(0.205) PCP3(0.205)
aa-ay	RH$ \to $I2(0.202) PO7(0.202) I1(0.201) **P1**(0.201) **P9**(0.201) PO2(0.200) P10(0.199) P2(0.199) PCP2(0.199) RF$ \to $CP1(0.206) CPz(0.206) CCP2(0.204) Cz(0.203) T7(0.203) CCP1(0.202) **P9**(0.202) **P1**(0.202) PCP1(0.202)
al-av	RH$ \to $Cz(0.196) CFC8(0.195) FC6(0.195) TP9(0.194) TP10(0.192) P10(0.192) PPO8(0.192) C4(0.192) RF$ \to $PCP3(0.212) CP1(0.212) P3(0.211) O1(0.210) OI1(0.209) P1(0.208) P5(0.207) P3P4(0.207)
al-aw	RH$ \to $FC5(0.206) FFC5(0.205) PCP4(0.204) C5(0.203) **CP2**(0.203) PCP5(0.202) **CP3**(0.202) FC3(0.202) P4(0.201) RF$ \to $P1(0.218) PCP3(0.215) **CP3**(0.213) **CP2**(0.212) PO3(0.212) P3(0.212) CP1(0.211) P5(0.209) PCP2(0.209)
al-ay	RH$ \to $Oz(0.219) PO1(0.217) OPO1(0.215) PO2(0.214) PPO1(0.213) PO3(0.213) PPO2(0.212) OI2(0.212) RF$ \to $O1(0.209) PPO6(0.206) PCP3(0.205) CP3(0.205) I1(0.205) PCP5(0.204) OI1(0.204) CP5(0.203)
av-aw	RH$ \to $P5(0.197) PCP7(0.197) **CCP5**(0.196) P9(0.195) **CP1**(0.195) FT7(0.195) PPO5(0.195) PCP5(0.194) P3(0.194) RF$ \to $**CCP5**(0.209) C3(0.204) CFC6(0.204) C1(0.204) FC6(0.202) **CP1**(0.202) CCP3(0.201) FC3(0.201) CP3(0.200)
av-ay	RH$ \to $I2(0.198) OPO1(0.196) I1(0.196) FAF5(0.194) PPO8(0.194) FT8(0.193) FFC7(0.193) OI1(0.193) RF$ \to $CCP8(0.205) T8(0.204) CP5(0.204) C6(0.202) FAF6(0.202) CFC6(0.202) CCP6(0.201) PCP8(0.201)
aw-ay	RH$ \to $PCP1(0.209) CCP3(0.207) PCP3(0.207) Pz(0.207) CP3(0.206) P1(0.206) CPz(0.206) P5(0.202) RF$ \to $T7(0.213) CFC7(0.210) OPO1(0.206) CCP7(0.204) P7(0.203) Oz(0.203) FT9(0.203) FT7(0.202)

## 3 Results and discussions

Table 1 summarises the sets of mostly coherent channels for all subject pairs. The channels are reported according to $\hbox{WC}\hbox{P}_N$ values in descending order. Each set consists of sixteen different channels from different areas of the brain, where the inter-subject sensorimotor oscillations are mostly associative in T–F space. The common channels which fall within selection criteria for both RH and RF are indicated as bold. Fig. 1 shows the selected channels (electrode montages) for subject pair al-aw, most of which lie around sensorimotor regions of the brain. Sensorimotor regions are highly responsive during MIs [25] and share associative inter-subject information.

Table 2 describes the classification accuracies achieved from inter-subject experiments. In *Case I*, available 118 channels are used to classify the MIs while only 16 channels, reported in Table 1, are used in *Case II*. Let's consider, $Acc\lpar S1 \to S2\rpar _{Case\; I}$ and $Acc\lpar S1 \to S2\rpar _{Case\; II}$ represent classification accuracies achieved from *Case I* and *Case II*, respectively. The trials from subject *S*1 are used to train and validate the classifier while the trials from *S*2 are used to test the classifier. $Acc\lpar S1 \to S2\rpar _{Case\; I} \lt Acc\lpar S1 \to S2\rpar _{Case\; II}$ indicates the increased classification performances of inter-subject BCI by using associative channels only. However, the maximum classification accuracy achieved for subject pair *aw-al* is $Acc\lpar aw \to al\rpar _{Case\; II} = 81.79\percnt $. But, the classification accuracy for this subject pair in *Case I* is $Acc\lpar aw \to al\rpar _{Case\; I} = 56.79\percnt $. Interestingly, $Acc\lpar aw \to al\rpar _{Case\; I} \ll Acc\lpar aw \to al\rpar _{Case\; II}$ evinces the potential applicability of the proposed channel selection method. Also, the classification performances are not symmetric, i.e. interchanging of training and testing trials significantly influences the performances. For example, $Acc\lpar al \to aw\rpar _{Case\; II} \ne Acc\lpar aw \to al\rpar _{Case\; II}$ and $Acc\lpar al \to aw\rpar _{Case\; I} \ne Acc\lpar aw \to al\rpar _{Case\; I}$ etc. The achieved classification accuracy $Acc\lpar al \to aw\rpar _{Case\; II} = 73.93\percnt $ is significantly lower than $Acc\lpar aw \to al\rpar _{Case\; II} = 81.79\percnt $. Such asymmetric *Acc* occurs, due to the fact that data-driven spatial filtering methods are prone to be overfitted while estimating unreliable parameters [26]. Although there are significant observations in which $Acc\lpar S1 \to S2\rpar _{Case\; I}\,{\rm \ll }\,Acc\lpar S1 \to S2\rpar _{Case\; II}$, some $Acc\lpar S1 \to S2\rpar $ do not necessarily show any promising performance in any of the *case*. The *cases* in which $Acc\lpar S1 \to S2\rpar _{Case\; I} \lt Acc\lpar S1 \to S2\rpar _{Case\; II}$ are italicised in Table 2.

**Table 2:** Classification accuracies (%): inter-subject BCI (The *cases* in which *Acc* (*S*1 → *S*2) _*Case I*_ < *Acc* (*S*1 → *S*2) _*Case II*_ are italicised.)

S1–S2	Case I	Case II	S1–S2	Case I	Case II
aa-al	60.71	*76.79*	al-aa	56.43	*57.14*
aa-av	56.79	*57.86*	av-aa	53.57	*61.07*
aa-aw	57.86	*65.36*	aw-aa	62.86	51.43
aa-ay	58.21	51.43	ay-aa	50.36	49.64
al-av	49.64	47.14	av-al	70.71	54.29
al-aw	69.64	*73.93*	aw-al	56.79	*81.79*
al-ay	63.57	*76.79*	ay-al	67.50	67.50
av-aw	55.71	52.14	aw-av	53.57	50.36
av-ay	62.14	57.50	ay-av	51.79	*53.21*
aw-ay	63.21	53.57	ay-aw	52.50	*64.29*
mean	59.75	*61.25*	mean	57.61	*59.07*

Many time variant psychophysiological factors including attention, memory load, spontaneous cognitive processes etc. [27] and user's basic characteristics, such as lifestyle, gender, age etc. influence the individual brain dynamics over time [28], thus affect BCI performance. This inherent fluctuation of individual brain dynamics causes inter-subject variability that poses difficulties while developing BCI without subject-specific calibration. In EEG-based BCI, undesired channels additionally degrade performances by producing outliers [18]. Fig. 2 shows both highly associative and dissociative inter-subject sensorimotor oscillations and their corresponding WC. In this Letter, a novel T–F approach has been proposed to sort out associative sensorimotor inter-subject channels while most of the literatures have addressed the adaptation of machine learning algorithms for compensating variabilities [13–17]. Results show that selecting inter-subject coherent channels can significantly increase performances, thus implicating future development of efficient inter-subject BCI paradigm. An interesting aspect that arises from the proposed approach is the possibility to generalise it towards finding common spatial components (i.e. a subspace of the data) instead of selecting individual electrodes; yet, this is left as a future endeavour.

## 4 Conclusions

Inter-subject BCI, without time-consuming, sometimes frustrating calibration session, seems more convenient to users. Nevertheless, inherent instantaneous variability in EEG signals poses significant challenges. Outliers that have been generated within insignificant channels may be due to diversity in psychophysiological or other factors, limit the development of BCI in subject independent context. Interestingly, the only channels which share similar sensorimotor dynamics can be employed for improving BCI performance. This study delineates a novel method for selecting inter-subject associative channels based on WC and show how BCI performances can be improved in subject independent settings.

## References

[1] Wang H. Chang W. Zhang C.: ‘Functional brain network and multichannel analysis for the P300-based brain computer interface system of lying detection’, Expert Syst. Appl., 2016, 53, pp. 117–128 (doi: 10.1016/j.eswa.2016.01.024)

[2] Farwell L. Richardson D. Richardson G.: ‘Brain fingerprinting classification concealed information test detects US Navy military medical information with P300’, Front. Neurosci., 2014, 8, p. 410 (doi: 10.3389/fnins.2014.00410) 25565941 10.3389/fnins.2014.00410 PMC4274905

[3] Ortiz Carreon F. Gonzalez Serna J. Montes Rendon A.: ‘Induction of emotional states in people with disabilities through film clips using brain computer interfaces’, IEEE Latin Am. Trans., 2016, 14, (2), pp. 563–568 (doi: 10.1109/TLA.2016.7437193)

[4] van de Laar B. Gurkok H. Plass-Oude Bos D.: ‘Experiencing BCI control in a popular computer game’, IEEE Trans. Comput. Intell. AI Games, 2013, 5, (2), pp. 176–184 (doi: 10.1109/TCIAIG.2013.2253778)

[5] Giedd J. Rapoport J.: ‘Structural MRI of pediatric brain development: what have we learned and where are we going?’, Neuron, 2010, 67, (5), pp. 728–734 (doi: 10.1016/j.neuron.2010.08.040) 20826305 10.1016/j.neuron.2010.08.040 PMC3285464

[6] Hasson U.: ‘Intersubject synchronization of cortical activity during natural vision’, Science, 2004, 303, (5664), pp. 1634–1640 (doi: 10.1126/science.1089506) 15016991 10.1126/science.1089506

[7] Abrams D. Ryali S. Chen T.: ‘Inter-subject synchronization of brain responses during natural music listening’, Eur. J. Neurosci., 2013, 37, (9), pp. 1458–1469 (doi: 10.1111/ejn.12173) 23578016 10.1111/ejn.12173 PMC4487043

[8] Jeannerod M.: ‘Mental imagery in the motor context’, Neuropsychologia, 1995, 33, (11), pp. 1419–1432 (doi: 10.1016/0028-3932(95)00073-C) 8584178 10.1016/0028-3932(95)00073-c

[9] Niazi I. Jiang N. Jochumsen M.: ‘Detection of movement-related cortical potentials based on subject-independent training’, Med. Biol. Eng. Comput., 2013, 51, (5), pp. 507–512 (doi: 10.1007/s11517-012-1018-1) 23283643 10.1007/s11517-012-1018-1 PMC3627050

[10] Ray A. Sitaram R. Rana M.: ‘A subject-independent pattern-based brain-computer interface’, Front. Behav. Neurosci., 2015, 9, p. 269 (doi: 10.3389/fnbeh.2015.00269) 26539089 10.3389/fnbeh.2015.00269 PMC4611064

[11] Rana M. Gupta N. Dalboni Da Rocha J.: ‘A toolbox for real-time subject-independent and subject-dependent classification of brain states from fMRI signals’, Front. Neurosci., 2013, 7, p. 170 (doi: 10.3389/fnins.2013.00170) 24151454 10.3389/fnins.2013.00170 PMC3798026

[12] Lu S. Guan C. Zhang H.: ‘Unsupervised brain computer interface based on intersubject information and online adaptation’, IEEE Trans. Neural Syst. Rehabil. Eng., 2009, 17, (2), pp. 135–145 (doi: 10.1109/TNSRE.2009.2015197) 19228561 10.1109/TNSRE.2009.2015197

[13] Samek W. Meinecke F. Muller K.: ‘Transferring subspaces between subjects in brain–computer interfacing’, IEEE Trans. Biomed. Eng., 2013, 60, (8), pp. 2289–2298 (doi: 10.1109/TBME.2013.2253608) 23529075 10.1109/TBME.2013.2253608

[14] Samek W. Kawanabe M. Muller K.: ‘Divergence-based framework for common spatial patterns algorithms’, IEEE Rev. Biomed. Eng., 2014, 7, pp. 50–72 (doi: 10.1109/RBME.2013.2290621) 24240027 10.1109/RBME.2013.2290621

[15] Lotte F. Guan C.: ‘Learning from other subjects helps reducing Brain-Computer Interface calibration time’. 2010 IEEE Int. Conf. on Acoustics, Speech and Signal Processing, Dallas, TX, 2010, pp. 614–617

[16] Devlaminck D. Wyns B. Grosse-Wentrup M.: ‘Multisubject learning for common spatial patterns in motor-imagery BCI’, Comput. Intell. Neurosci., 2011, 2011, pp. 1–9 (doi: 10.1155/2011/217987) 22007194 10.1155/2011/217987 PMC3191786

[17] Fazli S. Popescu F. Danóczy M.: ‘Subject-independent mental state classification in single trials’, Neural Netw., 2009, 22, (9), pp. 1305–1312 (doi: 10.1016/j.neunet.2009.06.003) 19560898 10.1016/j.neunet.2009.06.003

[18] Arvaneh M. Guan C. Ang K.K.: ‘Optimizing the channel selection and classification accuracy in EEG-based BCI’, IEEE Trans. Biomed. Eng., 2011, 58, (6), pp. 1865–1873 (doi: 10.1109/TBME.2011.2131142) 21427014 10.1109/TBME.2011.2131142

[19] Klein A. Sauer T. Jedynak A.: ‘Conventional and wavelet coherence applied to sensory–evoked electrical brain activity’, IEEE Trans. Biomed. Eng., 2006, 53, (2), pp. 266–272 (doi: 10.1109/TBME.2005.862535) 16485755 10.1109/TBME.2005.862535

[20] Cui X. Bryant D. Reiss A.: ‘NIRS-based hyperscanning reveals increased interpersonal coherence in superior frontal cortex during cooperation’, NeuroImage, 2012, 59, (3), pp. 2430–2437 (doi: 10.1016/j.neuroimage.2011.09.003) 21933717 10.1016/j.neuroimage.2011.09.003 PMC3254802

[21] Ramoser H. Muller-Gerking J. Pfurtscheller G.: ‘Optimal spatial filtering of single trial EEG during imagined hand movement’, IEEE Trans. Rehabil. Eng., 2000, 8, (4), pp. 441–446 (doi: 10.1109/86.895946) 11204034 10.1109/86.895946

[22] Svozil D. Kvasnicka V. Pospichal J.: ‘Introduction to multi-layer feed-forward neural networks’, Chemometr. Intell. Lab. Syst., 1997, 39, (1), pp. 43–62 (doi: 10.1016/S0169-7439(97)00061-0)

[23] Addison P.: ‘The illustrated wavelet transform handbook’ (Institute of Physics Publication, Bristol, UK, 2002)

[24] Grinsted A. Moore J. Jevrejeva S.: ‘Application of the cross wavelet transform and wavelet coherence to geophysical time series’, Nonlin. Processes Geophys., 2004, 11, (56), pp. 561–566 (doi: 10.5194/npg-11-561-2004)

[25] Zich C. Debener S. Kranczioch C.: ‘Real-time EEG feedback during simultaneous EEG–fMRI identifies the cortical signature of motor imagery’, NeuroImage, 2015, 114, pp. 438–447 (doi: 10.1016/j.neuroimage.2015.04.020) 25887263 10.1016/j.neuroimage.2015.04.020

[26] Sannelli C. Vidaurre C. Müller K.: ‘Ensembles of adaptive spatial filters increase BCI performance: an online evaluation’, J. Neural Eng., 2016, 13, (4), p. 046003 (doi: 10.1088/1741-2560/13/4/046003) 27187530 10.1088/1741-2560/13/4/046003

[27] GonÃğalves S. de Munck J. Pouwels P.: ‘Correlating the alpha rhythm to BOLD using simultaneous EEG/fMRI: inter-subject variability’, NeuroImage, 2006, 30, (1), pp. 203–213 (doi: 10.1016/j.neuroimage.2005.09.062) 16290018 10.1016/j.neuroimage.2005.09.062

[28] Ahn M. Jun S.: ‘Performance variation in motor imagery brain–computer interface: a brief review’, J. Neurosci. Methods, 2015, 243, pp. 103–110 (doi: 10.1016/j.jneumeth.2015.01.033) 25668430 10.1016/j.jneumeth.2015.01.033

